# The Abnormal Expression of miR-205-5p, miR-195-5p, and VEGF-A in Human Cervical Cancer Is Related to the Treatment of Venous Thromboembolism

**DOI:** 10.1155/2020/3929435

**Published:** 2020-08-08

**Authors:** Yuting Wang, Zegao Zhang, Pengcai Tao, Maimaitiyimin Reyila, Xiaoli Qi, Jie Yang

**Affiliations:** ^1^Department of Radiation Oncology, People's Hospital of Xinjiang Uyghur Autonomous Region, Urumqi, Xinjiang 830001, China; ^2^Xinjiang Medical University, Urumqi, Xinjiang 830054, China

## Abstract

**Background:**

Low molecular heparin (LWMH) therapy can prevent the occurrence of VTE in tumor patients and may have a direct antitumor effect. However, the expression pattern of VEGF-A and microRNAs was less reported in cervical cancer subjects who received concurrent chemoradiotherapy (CCRT) or received anticoagulant treatment with low molecular weight heparin (LWMH) after CCRT (CCRT+LWMH).

**Methods:**

In this study, 30 cervical cancer subjects treated with CCRT and 30 cervical cancer patients treated with CCRT+LWMH were enrolled. We screened five miRNAs (miR-15a-5p, miR-16-5p, miR-29a-3p, miR-195-5p, and miR-205-5p), which have multiple binding sites with VEGF-A and are highly expressed in serum of patients with cervical cancer, by RT-qPCR. The expression level of VEGF-A was also detected by RT-qPCR and ELISA. Statistical methods were used for difference and correlation analyses.

**Results:**

We observed the curative effect in the two treatment methods. In the CCRT group, the total effective rate was 60.00%, and in the CCRT+LWMT group, the total effective rate was 83.33% (*P* = 0.013, *χ*^2^ = 6.129). Additionally, the serum levels of VEGF-A in the CCRT+LWMH group were downregulated, relative to the CCRT group (*P* < 0.05), and VEGF-A in serum was significantly positively correlated with venous thromboembolism (VTE) (*r* = 2.134, *P* = 0.035). Only miR-205-5p and miR-195-5p were upregulated in CCRT+LWMH, relative to CCRT (*P* < 0.05). In serum of patients with cervical cancer after CCRT+LWMH treatment, there was no significant correlation between VEGF-A and miR-15a-5p (*r* = −0.132, *P* = 0.209), miR-16-5p (*r* = −0.205, *P* = 0.311), or miR-29a-3p (*r* = −0.029, *P* = 0.662), but VEGF-A was significantly negatively correlated with miR-195-5p (*r* = −0.396, *P* = 0.040) and miR-205-5p (*r* = −0.315, *P* = 0.032). Furthermore, VTE was also significantly negatively correlated with miR-195-5p (*r* = −0.412, *P* = 0.031) and miR-205-5p (*r* = −0.123, *P* = 0.044).

**Conclusion:**

These data revealed roles for VEGF-A and these miRNAs as potential biomarkers in cervical cancer patients with VTE, which exhibited usage potential in the treatment of venous thromboembolism.

## 1. Background

Venous thromboembolism (VTE) includes deep vein thrombosis (DVT) and pulmonary embolism (PE), which is based on a hypercoagulable state, vascular wall damage, and tumor compression vessels ([1]). VTE is a common complication and the second cause of death in patients with malignant tumors, second only to the tumor itself, and a malignant tumor is an important cause of VTE [2]. According to statistics, 20% of VTEs occur in patients with malignant tumors and about 20% of patients with malignant tumors can be diagnosed with VTE, but this result is as high as 50% at autopsy [3]. VTE can be the first clinical manifestation of cancer patients. About 10% of patients with primary thrombosis are diagnosed with malignant tumors several years after the thrombotic event, and about 40% of the patients had distant metastasis by the time of diagnosis [4]. According to different pathological types, the overall incidence of VTE in the first hospitalized adult malignant tumors ranged from 2.7% to 12.1% [5], and the highest incidence of several tissue sources were the brain, uterus, bladder, pancreas, and stomach. In the advanced patients with distant metastases, the incidence of VTE is 4-13 times higher than that of lesions [6]. Thus, VTE may be closely related to the occurrence and development of tumors.

Many epidemiological studies have demonstrated that anticoagulant therapy not only prevents the occurrence of VTE in cancer patients but also may have a direct antitumor effect, thereby inhibiting tumor metastasis and prolonging patient survival [7]. Low molecular heparin (LWMH) is currently a commonly used anticoagulant drug with a strong antithrombotic effect, long half-life, less bleeding side effects, and high safety; clinical application and research show that in addition to its anticoagulant effect, LWMH also has antiangiogenic and antitumor effects ([8]). It is well known that tumor angiogenesis is a necessary condition for tumorigenesis, growth, invasion, and metastasis. Moreover, the proliferation of vascular endothelial cells is a key link in the process of angiogenesis. Vascular endothelial growth factor (VEGF) is the most important proangiogenic factor ([9]). Furthermore, radiation can induce tumor cells to secrete vascular endothelial growth factor (VEGF), promote pathological neovascularization, and cause a hypoxic state of tumor cells [10]. Hypoxia can upregulate VEGF-A levels in the tumor microenvironment again, thus increasing the vicious circle [11]. More importantly, LMWH can inhibit the secretion of VEGF-A by different tumor cell lines, inhibit the proliferation of vascular endothelial cells, and thus inhibit tumor angiogenesis. Marchetti et al. [12] confirmed that LMWH can inhibit the migration of vascular endothelial cells and the formation of luminal structures. Moreover, it has been found that LMWH and its sulfated derivative S-NACH, in addition to inhibiting tumor growth, can also promote the uptake of chemotherapeutic drugs by tumor cells, which may improve tumor resistance [13].

Cervical cancer is one of the most common gynecological malignancies that seriously threaten women's health [14]. Chemotherapy alone was a risk factor for VTE, and there are many reports about the relationship between cervical cancer and thrombotic disease [15]. However, there are few studies on preventive anticoagulant therapy for cervical cancer patients. In the present study, we assessed the curative and anticoagulant effects of LWMH on cervical cancer patients who received concurrent chemoradiotherapy (CCRT). The levels of VEGF-A and microRNAs (miR-15a-5p, miR-16-5p, miR-29a-3p, miR-195-5p, and miR-205-5p), which have multiple binding sites with VEGF-A, were analyzed in the serum of cervical cancer patients, in order to verify if they could be valuable biomarkers for rapid diagnosis of cervical cancer, as well as promising therapeutic agents.

## 2. Materials and Methods

### 2.1. Patient Enrollment

The study population included 30 cervical cancer patients who received concurrent chemoradiotherapy (CCRT) and 30 cervical cancer patients who received anticoagulant treatment with low molecular weight heparin (LWMH) after CCRT (CCRT+LWMH) (Table 1) from 2017 to 2018 in People's Hospital of Xinjiang Uyghur Autonomous Region.

Inclusive criteria were as follows: (1) local advanced stage (IB2-IVa stage) cervical squamous cell carcinoma confirmed by histopathology; (2) Karnofsky score (KPS) > 70 points; (3) 18 years old < age < 75 years; (4) no other history of malignant tumors; (5) for the initial treatment, no previous antitumor treatment; (6) observation and follow-up for at least 2 years; and (7) those who signed informed consent.

Exclusion criteria were as follows: (1) the following anticoagulation contraindications—(i) recent central nervous system hemorrhage and intracranial or spinal cord high-risk hemorrhage, (ii) active hemorrhage (hemorrhage): 24-hour transfusion more than 2 units, (iii) chronic and clinical significance measurable bleeding > 48 hours, (iv) thrombocytopenia (platelets < 50000/mcL), (v) severe platelet dysfunction, (vi) large-scale surgery with high risk of bleeding recently, (vii) basic disease of coagulopathy, (viii) fall, and (ix) spinal anesthesia/lumbar puncture; (2) metastatic tumor; (3) history of other malignant tumors or any malignant tumor; (4) combination with other serious systemic diseases and inability to tolerate antitumor treatment; (5) those who received chemotherapy or surgery before concurrent radiotherapy and chemotherapy; (6) those who did not complete the planned treatment plan in this study for various reasons.

### 2.2. Ethical Approval

This study was approved by the ethics committee of People's Hospital of Xinjiang Uyghur Autonomous Region (KY2016052011). All procedures performed in studies involving human participants were in accordance with the ethical standards of the institutional and/or national research committee and with the 1964 Helsinki Declaration and its later amendments or comparable ethical standards.

### 2.3. Short-Term Efficacy

Short-term efficacy, according to solid tumor evaluation criteria (response evaluation criteria in solid tumors (RECIST)), is divided into complete remission (CR), partial remission (PR), stable disease (SD), and progressive disease (PD).

### 2.4. miRNA and VEGF-A mRNA Isolation and Real-Time Fluorescence Quantitative PCR (RTq-PCR) Analysis

Baseline data of all subjects, including general data and tumor staging, grading, and size, were recorded. Elbow vein blood samples (3 mL) of the patients on an empty stomach were obtained. RT-qPCR was used to test the level of VEGF-A, miR-15a-5p, miR-16-5p, miR-29a-3p, miR-195-5p, and miR-205-5p. The TRIzol reagent (Invitrogen, Carlsbad, CA, USA) was used for extracting total RNA. According to the manufacturer's instructions (TIANGEN BIOTECH, Beijing, China), a reverse transcription step was performed to obtain the first-strand cDNA of miRNA with the miRcute Plus miRNA First-Strand cDNA Synthesis Kit (TIANGEN BIOTECH, Beijing, China). The miRcute Plus miRNA qPCR Kit (SYBR Green Master Mix, TIANGEN BIOTECH, Beijing, China) was used for the quantitative analysis of gene expression in an ABI 7500 fluorescence quantitative PCR instrument (ABI, USA). The amplification involved a denaturation step at 95°C for 15 min, and amplification and quantification were repeated for 40 cycles at 95°C for 20 s and 60°C for 40 s. Primer sequences were as follows (5′-3′): VEGF-A, forward: ATCACGAAGTGGTGAAGTTC and reverse: TGCTGTAGGAAGCTCATCTC; miR-15a-5p, forward: TAGCAGCACATAATGGTTTGTG; miR-16-5p, forward: TAGCAGCACGTAAATATTGGCG; miR-29a-3p, forward: TAGCACCATCTGAAATCGGTTA; miR-205-5p, forward: CGGTCCTTCATTCCACCGGAG; and miR-195-5p, forward: TGCGCTAGCAGCACAGAAATA. The reverse primer for miRNA RT-qPCR was provided by the manufacturer in the qPCR kit (no. CD109): U6, forward: CTCGCTTCGGCAGCACA and reverse: AACGCTTCACGAATTTGCGT, and glyceraldehyde 3-phosphate dehydrogenase (GAPDH), forward: ATTGTTGCCATCAATGACCC and reverse: AGTAGAGGCAGGGATGATGT. The data of the relative gene expression levels were calculated by the 2^−*ΔΔ*Ct^ method and presented as the fold change of transcripts for genes. GAPDH and U6 were used as internal controls for normalization in RT-qPCR.

### 2.5. Serum VEGF-A Level Detection with the Enzyme-Linked Immunosorbent Assay (ELISA)

VEGF-A level in serum was detected with the commercial human VEGF-A ELISA Kit (Abcam, Cambridge, MA) following the procedures provided by the manufacturer. The serum sample was added to each well, and then, the antibody mixture was added for incubation. After that, the wells were washed to remove unbound substances. Subsequently, the tetramethylbenzidine substrate was added and incubated at room temperature. After adding the stop solution, the intensity was detected with a microplate reader (BioTek, Winooski, USA) at 450 nm wavelength.

### 2.6. Analysis and Statistics

Statistical data were analyzed by SPSS 17.0 software (IBM SPSS, Inc., Chicago, IL, USA), and the analysis of counting data was performed by the *χ*^2^ test. The comparison of the mean of measurement data was performed by the Student *t*-test. The Spearman correlation coefficient was used for correlation analysis of expression levels of these miRNAs and VEGF-A and VTE. The difference was considered statistically significant at *P* < 0.05.

## 3. Results

### 3.1. The Clinical Features of the Cervical Cancer Patients

A total of 60 cases of cervical cancer patients were received to be treated with CCRT+LWMH or CCRT. The clinical and pathological features are shown in Table 1.

### 3.2. Observation of the Curative Effect

The curative effect of 60 patients with cervical cancer was compared after treatment with CCRT or CCRT+LWMH. In the CCRT group, 1 case had clinical complete response (CR), 17 cases had clinical partial response (PR), 12 cases had no change in disease (NC), and no case (0 case) had progressive disease (PD); the total effective rate was 60.00%. In the CCRT+LWMH group, 7 patients had complete clinical remission (CR), 18 patients had clinical partial remission (PR), 5 patients had no change in disease (NC), and no patient (0 patient) had progressive disease (PD). The total effective rate was 83.33%. The difference between the two was statistically significant (*P* = 0.013, *χ*^2^ = 6.129) (Table 2).

### 3.3. The Expression of VEGF-A in the Serum of Patients with Cervical Cancer Is Correlated with VTE after CCRT+LWMH Treatment

**Figure 1:**
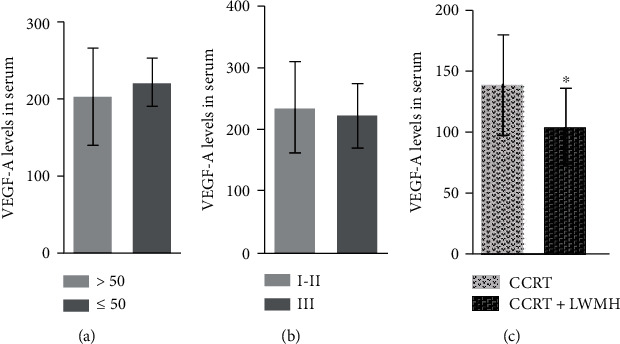
The expression levels of VEGF-A are tested by using RT-qPCR in serum of patients with cervical cancer. (a) The expressed changes of VEGF-A mRNA in different age groups. (b) The expressed changes of VEGF-A mRNA in different stages. (c) The expressed changes of VEGF-A mRNA after different treatments (^∗^*P* < 0.05 vs. CCRT). CCRT: concurrent chemoradiotherapy. CCRT+LWMH: low molecular weight heparin (LWMH) treatment after CCRT.

The changes of VEGF-A levels in the serum were measured by ELISA. Firstly, age and clinical grade had no significant effect on VEGF-A levels (*P* > 0.05, Figures 1(a) and 1(b)). Furthermore, compared with the CCRT group, the levels of VEGF-A in the CCRT+LWMH group were downregulated significantly (*P* < 0.05, Figure 1(c)). The correlation between VEGF-A and VTE in patients with cervical cancer was assessed. In serum of patients with cervical cancer after CCRT+LWMH treatment, the level of VEGF-A was significantly positively correlated with VTE (*r* = 2.134, *P* = 0.035).

### 3.4. CCRT+LWMH Treatment Changes the Levels of miR-205-5p and miR-195-5p in the Serum of Patients with Cervical Cancer after CCRT

**Figure 2:**
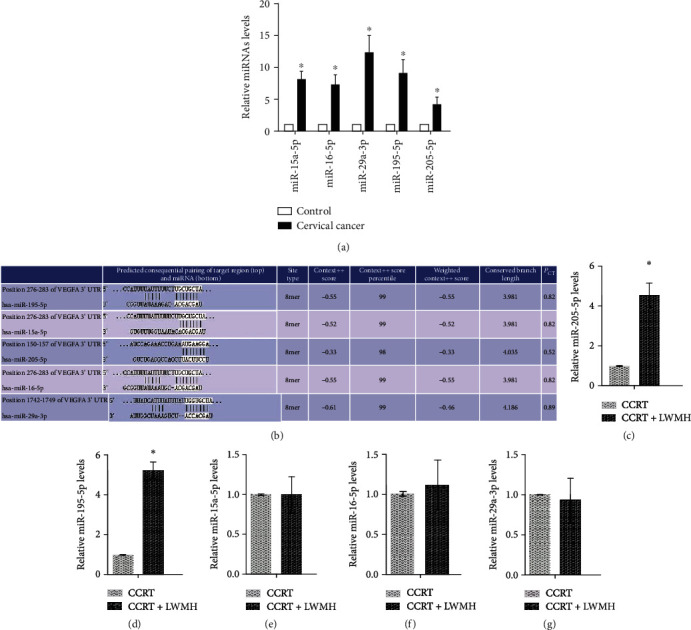
The expression levels of miR-15a-5p, miR-16-5p, miR-29a-3p, miR-195-5p, and miR-205-5p are tested by using RT-qPCR in serum of patients with cervical cancer: (a) levels of miRNAs in control and cervical cancer groups (^∗^*P* < 0.05 vs. control); (b) the binding sites between miRNAs and VEGF-A; (c–g) the expressed changes of the miRNAs after different treatments (^∗^*P* < 0.05 vs. CCRT). CCRT: concurrent chemoradiotherapy. CCRT+LWMH: low molecular weight heparin (LWMH) treatment after CCRT.

Furthermore, we screened five miRNAs (miR-15a-5p, miR-16-5p, miR-29a-3p, miR-195-5p, and miR-205-5p), which have multiple binding sites with VEGF-A and are highly expressed in serum of patients with cervical cancer (Figures 2(a) and 2(b)). Then, we observed the level changes of miR-15a-5p, miR-16-5p, miR-29a-3p, miR-195-5p, and miR-205-5p after treatment. It was shown that the levels of miR-205-5p and miR-195-5p were upregulated in CCRT+LWMH (*P* < 0.05, Figures 2(c) and 2(d)) compared to CCRT, but there were no significant differences of miR-15a-5p, miR-16-5p, and miR-29a-3p levels between CCRT and CCRT+LWMH (*P* > 0.05, Figures 2(e)–2(g)).

### 3.5. Correlations between the miRNAs and VEGF-A in Patients with Cervical Cancer after CCRT+LWMH Treatment

In serum of patients with cervical cancer after CCRT+LWMH treatment, there was no significant correlation between VEGF-A and miR-15a-5p (*r* = −0.132, *P* = 0.209), miR-16-5p (*r* = −0.205, *P* = 0.311), or miR-29a-3p (*r* = −0.029, *P* = 0.662). The serum level of VEGF-A was significantly negatively correlated with miR-195-5p (*r* = −0.396, *P* = 0.040) and miR-205-5p (*r* = −0.315, *P* = 0.032) (Table 3).

### 3.6. Correlations between the miRNAs and VTE in Patients with Cervical Cancer

In patients with cervical cancer after CCRT+LWMH treatment, there was no significant correlation between VTE and miR-15a-5p (*r* = −0.258, *P* = 0.096), miR-16-5p (*r* = −0.119, *P* = 0.074), or miR-29a-3p (*r* = −0.421, *P* = 0.138). But VTE was significantly negatively correlated with miR-195-5p (*r* = −0.412, *P* = 0.031) and miR-205-5p (*r* = −0.123, *P* = 0.044) (Table 4).

## 4. Discussion

In the present study, CCRT+LWMH treatment could effectively slow the progress of the VTE and decrease the serum VEGF-A levels in cervical cancer patients. Moreover, miR-195-5p and miR-205-5p, which have binding sites with VEGF-A and are highly expressed in serum of patients with cervical cancer, were also upregulated after CCRT+LWMH treatment. Furthermore, VTE was remarkably correlated with VEGF-A, miR-195-5p, and miR-205-5p. Our findings suggested that the abnormal expression of miR-205-5p, miR-195-5p, and VEGF-A in human cervical cancer is related to the VTE.

VTE is a recognized complication of cervical cancer and has high morbidity and mortality in cervical cancer patients ([16]). However, the pathophysiological mechanisms of VTE in patients with cervical cancer are complex, which include activation of coagulation and fibrinolysis systems, inflammatory response, acute-phase response, apoptosis, and cytokine production ([17]). Many epidemiological studies have demonstrated that anticoagulant therapy not only prevents the occurrence of VTE in cancer patients but also may have a direct antitumor effect, thereby inhibiting tumor metastasis and prolonging patient survival [18]. LWMH is currently a commonly used anticoagulant drug with strong antithrombotic effect, long half-life, less bleeding side effects, and high safety [19]. In the present study, in the course of treatment for remission of VTE, we found that the total effective rate was 83.33% in CCRT with LWMH treatment, but the total effective rate was only 60% in CCRT without LWMH treatment. Our study confirmed that LWMH injection could be an efficient way to alleviate VTE with CCRT treatment.

The VTE-related molecular mechanisms were further investigated. It was found that tumor cells can produce tissue factor (TF), cancer procoagulant (CP), inflammatory factors, and cytokines to directly activate blood coagulation [20]. Studies [21] have shown that overexpression of TF is a major factor in the development of cancer-associated VTE. TF not only activates the exogenous coagulation cascade but also stimulates tumor angiogenesis via upregulating VEGF and downregulating thrombospondin [22]. Importantly, it has been reported that LMWH can inhibit the secretion of VEGF by different tumor cell lines, inhibit the proliferation of vascular endothelial cells, and thus inhibit tumor angiogenesis ([23]). Consistent with the above studies, we confirmed that LMWH inhibits the serum levels of VEGF-A from cervical cancer patients. VEGF-A, a key subtype of VEGF which is a member of the platelet-derived growth factor (PDGF) superfamily, is produced and secreted by a variety of cells and can promote vascular hyperplasia and regulate vascular permeability under both physiological and pathological conditions [24]. Waltham et al. [23] found that VEGF can promote the lumen recanalization and thrombus organization. Similarly, overexpression of VEGF-A could inhibit the thrombotic occlusion and incidence of mural thrombus in a balloon injury model of the femoral artery of New Zealand white rabbits [25]. These results suggested that the abnormal expression of VEGF-A plays a key role in the thrombogenesis.

VEGF and its functions have been confirmed to be regulated by different miRNAs. For example, the serum levels of VEGF in transient and acute ischemic stroke patients were downregulated compared to control which was associated with the expression levels of miR-195-5p [26], and miR-195-5p and miR-451a have been shown to target VEGF-A in several experimental settings [27–29]. Additionally, miR-205-5p suppressed VEGF expression in human keloid fibroblasts [30]. Moreover, a number of studies focus on the role of miRNA-mediated VEGF in cervical cancer. It was reported that miR-144 inhibits growth and metastasis of cervical cancer cells by suppressing VEGF-A and VEGF-C [31]. In the present results, we also verified that the abnormal expression of miR-205-5p and miR-195-5p in the serum of cervical cancer patients was negatively related to serum VEGF-A.

Interestingly, both miR-195-5p and miR-205-5p levels were significantly upregulated in CCRT+LWMH-treated patients compared to the CCRT group. This elevation could be related to the VET event during cervical cancer development, especially when associated with lower VEGF-A serum levels. Although we cannot discern the exact time point at which miR-195-5p and miR-205-5p were upregulated, the negative relation between the two miRs and VEGF-A may be used to mark when the VTE happens or is cured. In patients with cervical cancer, the present results found that VTE was significantly negatively correlated with miR-195-5p and miR-205-5p. We have predicted that VEGF-A could be a target gene of miR-195-5p or miR-205-5p. Previous studies have identified the hypothesis in different cell lines, and a negative correlation between miR-195-5p/miR-205-5p and VEGF was demonstrated [32, 33].

Our studies suggest a role for VEGF-A, miR-195-5p, and miR-205-5p as potential biomarkers in cervical cancer patients treated for venous thromboembolism.

## 5. Limitations

This study does not further discuss why the VET was positively correlated with VEGF-A or negatively related to miR-195-5p/miR-205-5p. Our data were collected in a small group of patients and at a single institution.

## Figures and Tables

**Table 1:** Clinical and pathological features.

	Cases		
	Total	CCRT	CCRT+LWMH
Age (years)			
>50	39	20	19
≤50	21	10	11
Gender			
Female	60	30	30
Alcohol			
Yes	24	11	13
No	36	19	17
Cigarette^a^			
Yes	6	3	3
No	52	26	26
Clinical stage			
I-II	33	17	16
III	27	12	15

^a^Some patients were not in either of the subgroups due to undetermined record.

**Table 2:** Comparison of short-term efficacy.

Groups	Case	CR	PR	NC	PD	CR+PR
CCRT	30	1	17	12	0	60.00%
CCRT+LWMH	30	7	18	5	0	83.33%
Total	60	8	35	17	0	

**Table 3:** Correlation between the miRNAs and VEGF-A in patients with cervical cancer after CCRT+LWMH treatment.

	VEGF-A	
	*r*	*P*
miR-15a-5p	-0.132	0.209
miR-16-5p	-0.205	0.311
miR-29a-3p	-0.029	0.662
miR-195-5p	-0.396	0.040
miR-205-5p	-0.315	0.032

**Table 4:** Correlation between the miRNAs and VTE in patients with cervical cancer after CCRT+LWMH treatment.

	VTE	
	*r*	*P*
miR-15a-5p	-0.258	0.096
miR-16-5p	-0.119	0.074
miR-29a-3p	-0.421	0.138
miR-195-5p	-0.412	0.031
miR-205-5p	-0.123	0.044

## Data Availability

The data used to support the findings of this study are available from the corresponding author upon request.
